# Sleeping Beauty Mouse Models of Cancer: Microenvironmental Influences on Cancer Genetics

**DOI:** 10.3389/fonc.2019.00611

**Published:** 2019-07-09

**Authors:** Amy Guimaraes-Young, Charlotte R. Feddersen, Adam J. Dupuy

**Affiliations:** Department of Anatomy and Cell Biology, Roy J. and Lucille A. Carver College of Medicine, University of Iowa, Iowa City, IA, United States

**Keywords:** sleeping beauty, insertional mutagenesis, mouse models of cancer, cancer, genetics

## Abstract

The Sleeping Beauty (SB) transposon insertional mutagenesis system offers a streamlined approach to identify genetic drivers of cancer. With a relatively random insertion profile, SB is uniquely positioned for conducting unbiased forward genetic screens. Indeed, SB mouse models of cancer have revealed insights into the genetics of tumorigenesis. In this review, we highlight experiments that have exploited the SB system to interrogate the genetics of cancer in distinct biological contexts. We also propose experimental designs that could further our understanding of the relationship between tumor microenvironment and tumor progression.

## Utilization of Sleeping Beauty to Model Tumor Formation in Mice

Sleeping Beauty (SB) is a two-part DNA transposon system that has become an integral tool in identifying genetic drivers of cancers in mouse models. SB-induced cancer models have led to the discovery of novel cancer genes while comparative genomics have demonstrated the relevance of these models to human disease (1–8). In this review, we highlight characteristics of SB that make it uniquely equipped for unbiased *in vivo* forward genetic cancer screens and describe various ways in which SB cancer models are being used to address the impact of tumor microenvironment on cancer biology. Such approaches have provided insight into the multifaceted interplay between somatic mutations and other exposures that drive tumor progression. Collectively these experiments highlight the flexibility of SB mutagenesis in addressing complex genetic questions.

### SB Origins and Optimization for Forward Genetic Screens

SB is derived from the Tc1/mariner superfamily of “cut-and-paste” transposable elements widely encoded across all animal kingdoms but silenced in vertebrates by the evolutionary accumulation of mutations. Site-directed mutagenesis of a consensus DNA sequence derived from fish species permitted synthesis of the active transposase enzyme and flanking recognition sequences mobilized by the enzyme (the transposon) (9). Functionality was found to be preserved in *trans* as a two-part system when co-transfection of two plasmids, one containing the transposase and the other containing a selectable cassette flanked by the transposase recognition sequences, resulted in successful integration of the selection cassette within chromosomal DNA (9). Indeed, the SB system demonstrated superior activity in mammalian cells relative to other transposons tested and, in 2001, several groups reported germline transmission and chromosomal transposition of SB in mice harboring both the transposon and transposase in their genomes (i.e., double transgenic mice) (9–12). For use in forward genetic cancer screens, the SB system needed to be able to serve as an effective mutagen and achieve a mutational frequency sufficient to induce tumors.

**Figure 1:**
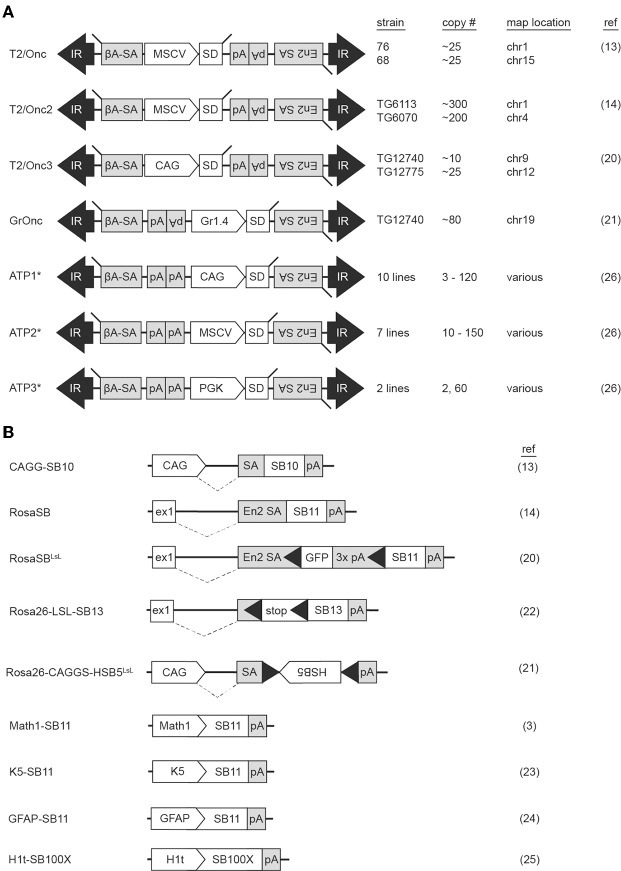
Multiple Sleeping Beauty constructs have been engineered into mouse lines for use in forward genetic screens. Mouse strains harboring transposon **(A)** or transposase **(B)** constructs are available for use in forward genetic cancer screens. Transposition is initiated when a mouse harboring a transposon concatemer is crossed with a mouse harboring a transposase. For most transposon constructs, multiple lines have been developed, each harboring a different number of transposons on a different chromosome **(A)**. Transposase constructs incorporate various modifications to regulate location of enzyme activation **(B)**. ßA-SA, beta-actin splice acceptor; CAG, CAG promoter; En2 SA, engrailed-2 splice acceptor; ex1, Rosa26 exon 1; Gr1.4, Graffi1.4 murine leukemia virus LTR; IR, inverted repeat; MCSV, murine stem cell virus promoter; pA, polyadenylation signal; PGK, phosphoglycerate kinase promoter; SD, splice donor. Black triangles within transposase constructs represent Lox sequence sites recognized by Cre recombinase. ^*^The ATP constructs incorporate both Sleeping Beauty and PiggyBac transposons.

The first SB transposons successfully used in insertional mutagenesis screens were T2Onc and T2Onc2 (13, 14). The constructs are similar: both contain the murine stem cell virus long terminal repeat (MSCV-LTR) promoter and a splice donor (SD) cassette to drive ectopic overexpression of downstream exons (mimicking oncogenic gain of function mutations). Gene trap components consisting of splice acceptors (SAs) on both DNA strands and a bidirectional polyadenylation signal (pA) to prematurely terminate gene transcription (mimicking tumor suppressor loss of function mutations) were also engineered into the constructs, permitting gene disruption independent of transposon reinsertion orientation (Figure 1A). Relative to T2Onc, T2Onc2 contains a larger SA fragment and an overall smaller size to increase transposition rate (14). Overall, the introduction of these components into the SB construct increased the likelihood of transcriptional interference upon reintegration of the mobilized transposon into gene-encoded chromosomal DNA.

**Figure 2:**
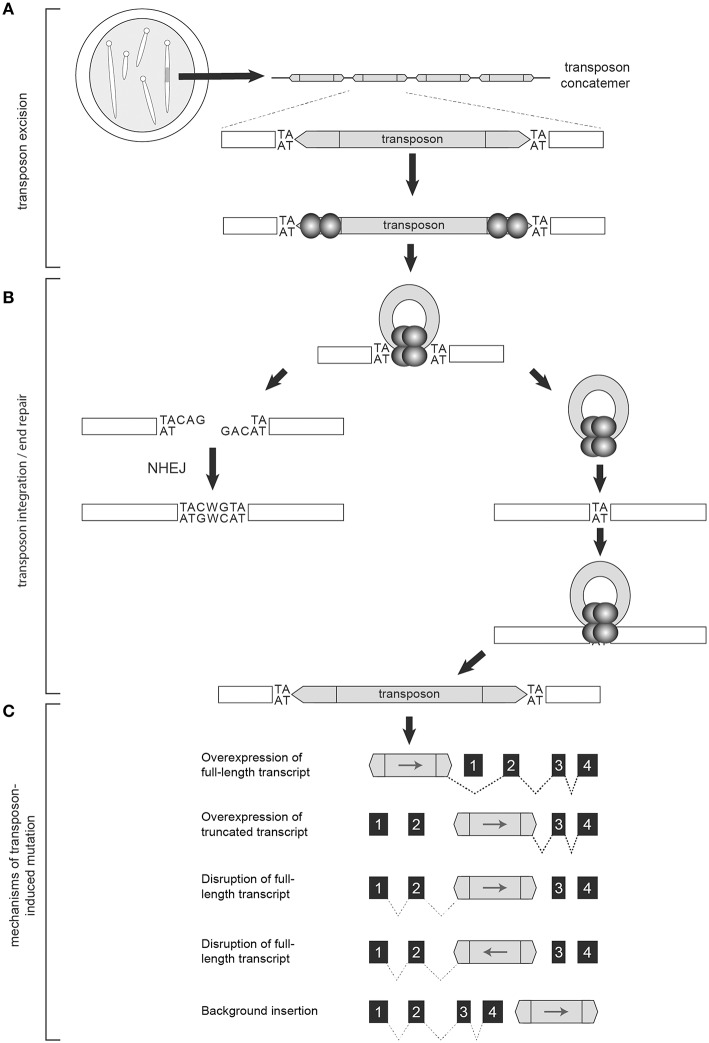
The Sleeping Beauty System is designed to disrupt gene expression. Transgenic mice used in forward genetic SB screens harbor a transposon concatemer in one of their chromosomes **(A)**. Both ends of the transposon contain recognition sites that are necessary for transposon activation by the transposase (represented by the spherical structures) **(A)**. Upon mobilization, the remaining DNA is preferentially repaired by non-homologous end joining (NHEJ) while the mobilized transposon integrates into a TA-dinucleotide site within the parent genome **(B)**. Depending on location and orientation of insertion, the gene trap components of the transposon are capable of altering transcription to either increase or disrupt gene expression, resulting in oncogenic activation, or tumor suppressor disruption, respectively **(C)**.

Generation of the T2onc and T2Onc2 transgenic mice by pronuclear injection of linearized plasmid DNA yielded offspring with multiple copies of adjoining transposons, a phenomenon caused by homologous recombination between constructs, and subsequent concatemerized integration into chromosomal DNA (Figure 2A) (15). The result was an increased number of insertional events per cell when crossed with a transgenic mouse with an activating SB transposase (SBase) construct. T2Onc transgenic lines are “low copy number,” with ~25 transposons per cell; T2Onc2 lines contain a “high copy number” of ~150–300 transposons per cell (13, 14). While transposon copy number contributes to increased frequency of transposon mutagenesis, alterations to the SBase component of the system also influence efficiency.

The first transgenic SBase mouse contained the originally identified transposase sequence (SB10) under the control of the chimeric chicken beta-actin and cytomegalovirus immediate early promoter sequences (CAGG) promoter (Figure 1B) (9, 13). This promoter was selected for its ubiquitous activity in mice (16). A shortcoming of transgenic models, however, is transgene silencing via methylation secondary to both concatemerization and positional effects depending on the chromosomal region into which the transgene inserts. This was of particular concern, as *in vitro* work demonstrated the influence of transposase expression levels on transposition rate (17). Indeed, transposition frequency in the double-transgenic low-copy T2Onc; *CAGGS*-*SB10* and high-copy T2Onc2; *CAGGS*-*SB10* mice were insufficient for tumorigenesis on a wildtype background, with transposase levels found to be quite low (13, 18). To address this, a knock-in SBase mouse (*RosaSB11*) was engineered by targeting an optimized transposase sequence (referred to as SB11) into the *Gt(ROSA)26Sor* locus (14). *SB11* is similar to *SB10* with the exception of several amino acid substitutions found to increase transposition activity (17). The *Gt(ROSA)26Sor* locus was selected as prior research had demonstrated ubiquitous expression of genetic constructs targeted to this region (19). Crossing of the *RosaSB11* mouse to either the high-copy T2Onc2 or low-copy T2Onc mouse resulted in transposition-induced cancer with a predilection for lymphoid leukemias (14, 18). Interestingly, the low-copy T2Onc; *RosaSB11* mice demonstrated decreased embryonic lethality and a prolonged latency relative to the high-copy T2Onc2; *RosaSB11* offspring (18). Indeed, these early experiments demonstrated the ability of both components of the SB system to influence *in vivo* cancer models.

Since these initial publications, other mice have been engineered to expand the functionality of SB insertional mutagenesis, with 16 distinct SB constructs used to-date in *in vivo* cancer screens (Figures 1A,B). One limitation of the T2Onc2 mouse was its predisposition for leukemias despite ubiquitous SB activation in the animal (14). A possible explanation for this phenotype was the promoter used to drive oncogenic over-expression of downstream exons upon transposon reinsertion. Indeed, the MSCV-LTR promoter demonstrates greatest transcriptional activity in hematopoietic cells, potentially biasing the mutagenic rate in favor of blood tumors (20). To address this, the T2Onc3 transposon was generated by substituting the MSCV-LTR promoter for the CMV enhancer/chicken beta-actin (CAG) promoter (20). The T2Onc3 mouse demonstrated increased SB expression in epithelial cells and the formation of various solid tumors (20). Another limitation of the SB system was the high rate of embryonic lethality due to constitutive transposition induced by early, ubiquitous activation of the *RosaSB* transposase (14). As such, a *LoxP-stop-Lox* cassette was incorporated into the *RosaSB* allele (*RosaSbas*e^*LSL*^) rendering a *Cre*-inducible transposase (20). For the first time, SB activation could be induced in a tissue-specific manner by breeding with any of the *Cre*-transgenic mouse lines. Other SB design modifications have included the introduction of different transposon promoters, tissue-specific promoters for selective transposase activation, and transposase constructs with increased activity (3, 21–25). Mice harboring fusion constructs capable of mobilization by either SBase or the PiggyBac (PB) transposase (referred to as activating/inactivating transposons, or “ATPs”) have also been generated (26). Collectively these models demonstrate the versatility of the SB system to model cancers.

### Regulation of the SB Machinery

Mobilization of the SB transposon is a regulated process influenced by differences in SBase binding affinities, host cell factors, and DNA methylation status (recently reviewed elsewhere) (27). The transposase harbors both a catalytic and DNA binding domain, which is divided into two DNA binding subdomains, RED and PAI (28). Briefly, a pair of direct repeat (DR) binding sites in both inverted terminal repeat (ITR) ends of the transposon (collectively referred to as IR/DR elements) serve as transposase recognition sites for mobilization by the transposase. All four of these sites must be present for SB transposition to occur (28). Modifications to the primary DNA sequence of the ITR and/or transposase can influence transposition frequency (17, 29, 30).

In mammalian cells, the transcription factor HMG2L1 (also referred to as HMGXB4) aids transposase expression (31). Once transcribed, the transposase protein actively recruits the host cell High Mobility Group protein (HMGB1) to enhance its binding to an inner DR and facilitate DNA bending and complex formation with the inner DR of the ITR at the opposite end of the transposon (32). CpG methylation of transposon concatemers induces a condensed heterochromatin structure that further enhances this protein-DNA synaptic complex formation (33). Transposon excision then proceeds via DNA double strand breaks (32, 34). The transposase recruits Ku70 and DNA-dependent protein kinases that preferentially repair the excision sites via non-homologous end joining (NHEJ) (35). The transcription factor Miz-1 is also recruited, leading to down-regulation of Cyclin D1 and cell cycle arrest in G1, a phase favoring NHEJ (36). Upon repair, a 7-base footprint remains (Figure 2B) (35, 37). The exposed 3′-OH groups on the ends of the excised transposons are used in the transfer reaction upon transposon reintegration (9).

SB transposons reintegrate into chromosomal DNA at TA-dinucleotide sites with preference for AT-repeat rich sequences, presumably due to optimized DNA bendability (Figure 2B) (9, 38). Additional TA dinucleotide sequences are added on either end of the integrated transposon upon repair (9). Reintegration is highly efficient, with rates in murine embryonic stem cells as high as 75% (39, 40). There is some debate regarding the integration bias of the SBase enzyme. Several studies of unselected insertion events detected no significant bias for insertion near specific chromatin marks or genomic features (40, 41). However, another report did find a slight bias for SB insertion events within transcribed regions (42). One clear source of bias occurs when SB is activated from within chromosomes resulting in the “local hopping” phenomenon—the tendency of SB transposons to reintegrate into the original donor chromosome (43). This bias can be removed by excluding reintegration sites that land within the donor chromosome from analyses.

### Other Integrating Vector Systems

Other integrating vector systems commonly used in mouse models of cancer include retroviruses and the PiggyBac (PB) transposon system. Retroviral mutagenesis has the longest history of use in forward genetic cancer screens, however several drawbacks exist (44, 45). The cellular tropism of retroviruses used in mouse models confines them to the production of mammary and hematopoietic tumors (46, 47). These models also demonstrate a bias for oncogene activation over tumor suppressor inactivation (48, 49). Causative gene identification can be difficult due to the ability of retroviral enhancers to influence expression of genes hundreds of kilobases away from their integration sites (50, 51). Moreover, non-random integration patterns are commonly observed, with murine leukemia virus (MLV) exhibiting bias for transcription start sites (TSSs) of transcriptionally active genes (52). Similar to SB, PB is a “cut-and-paste” transposon with a unique integration motif (“TTAA” vs. “TA” for SB) that demonstrates activity across tissue types and is capable of candidate oncogene and tumor suppressor identification (41, 53, 54). However, as with retroviruses, PB shows preferential bias for TSSs and actively transcribed regions (55). Researchers have suggested combining SB and PB screens to maximize candidate tumor driver identification which would be readily feasible using mice harboring an ATP transposon (Figure 1A) (56). Indeed, in a model of pancreatic cancer using ATP1 activated by PB transposase (PBase), a rare subtype of pancreatic cancer not identified in two distinct models of SB-induced pancreatic cancer was observed (22, 57, 58). Currently, only limited reports are available on candidate tumor drivers identified in dual PB/SB screens in combination with PBase or SBase (56). It remains to be seen the extent to which these two systems may complement each other in the quest for tumor driver identification.

### Interpreting SB Data

SB-induced tumors are harvested and the genomic DNA extracted. Since the transposon also functions as a “tag,” mutated sites within DNA are easily identified. Candidate tumor drivers (historically referred to as Common Insertion Sites or “CISs”) are called when a gene harbors integrations at a higher frequency than expected by chance alone. Put another way, SB insertions facilitating proliferation and malignant transformation are selected for and clonally expanded, resulting in non-random mutation profiles. To identify candidate tumor drivers, SB-genomic DNA junctions must be amplified, sequenced, mapped, and statistically evaluated. Variations at any step of the process can influence sensitivity, quantification, and gene identification. This complicates comparisons between independent studies.

After tumor harvesting and DNA extraction, the tumor DNA is fragmented. Initial studies accomplished this using restriction enzyme digestion (59). DNA shearing via sonication is now preferred, however, as it results in uniformly sized fragments. SB-DNA junctions are then amplified using linker-mediated PCR (59–61). Next generation sequencing technology permits the simultaneous sequencing of products and has dramatically increased the ability to identify SB insertional events. Platforms commonly utilized today include the Roche 454 GS FLX Pyrosequencer (with an average read depth per sample in the thousands) and Illumina (with an average read depth per sample in the 100,000s). The combination of DNA fragmentation via sonication, which greatly reduces PCR amplification bias due to size variability, with the high read depths achievable on next generation sequencing platforms (primarily Illumina) permits semi-quantification of transposon reads (55, 59–61).

Upon read acquisition, sequences are aligned and mapped to the mouse genome (62). SB insertions mapping to the chromosome harboring the SB transposon concatemer are omitted from downstream analyses to account for local hopping. Consequently, studies utilizing only one SB mouse line do not achieve full genome coverage and genes facilitating tumorigenesis residing on the parent chromosome are missed. To overcome this limitation, investigators may elect to use two SB mouse lines in a screen. Omission of sequence reads mapping to the parent chromosome of one line are included in the reads from the other line and vice versa. It is important to note that mapping errors can result in erroneous candidate gene identification. As an example, *Sfi1* was identified as a candidate tumor driver in numerous screens, but was subsequently found to be a false call due to inaccurate mapping of reads to repetition-rich regions of the genome (63). It is inevitable that algorithms used to interpret the vast amount of genetic data generated from SB screens will continue to improve.

Various statistical models have been used to identify candidate tumor drivers among the mapped SB-DNA reads including: Monte Carlo (MC) simulation, Gaussian Kernel Convolution (GKC) method, two-dimensional Gaussian Kernel Convolution method (2DGKC), Poisson distribution, Poisson Regression Insertion Model (PRIM), and gene-centric common insertion site (gCIS) analysis (44, 60, 64–67). MC, GKC, and Poisson-based methods take into account the local density of TA dinucleotides (i.e., potential SB insertion sites) within genomic intervals of user-defined size to localize clusters of disruptions (44, 64, 67). The 2DGKC and PRIM methods incorporate parameters permitting identification of possible co-occurring mutations within individual tumors (65, 66). A limitation to these approaches is that identifying biologically meaningful windows can be challenging. In contrast, the gCIS approach examines transcribed regions of the genome and determines an expected SB insertion rate for each RefSeq gene based on the number of TA sites within the RefSeq unit and 10 kilobases of promoter sequence (60). Overlap in candidate tumor driver calls among statistical methods ranges from 60–90%, however the gCIS approach has been shown to have increased sensitivity relative to MC and GKC methods (60, 68). In addition, the pattern of insertion can predict the role the candidate gene may have in tumorigenesis. Regions of integration with the majority of transposons oriented upstream of a gene with the transposon promoter (e.g., MSCV, CAG) in the same direction as gene transcription often leads to increased oncogene expression. Insertions throughout the length of a gene with no orientation bias are suggestive of decreased gene expression, or loss of tumor suppressor activity (Figure 2C).

More recently, RNA from SB tumors has been used to further assess SB-induced genetic alterations (69). In their methods paper, investigators evaluated RNA sequencing (RNA-Seq) data in juxtaposition with traditional DNA insertion site data obtained from the same SB tumors. Regions of the genome with candidate insertion sites had observable changes in transcript levels when compared to tumors lacking those same insertions. Transposon-RNA fusion transcripts were also identified, many of which corresponded with DNA-derived data (53 and 71% of fusions were supported by restriction enzyme digested LM-PCR and sheared LM-PCR methods, respectively). Additionally, novel fusions and alterations in transcript expression not readily ascertained from genomic data were also identified (69). Overall, transcript analyses permitted direct identification of genes altered by SB and the nature of the disruption (i.e., increased expression, decreased expression, formation of a truncated transcript, etc.). While it is not uncommon for researchers to amplify individual fusion products to further investigate SB-induced alterations, large scale transcriptome analyses have hitherto not been performed. Given its utility, it is likely that the RNA-Seq technique will be incorporated into future SB forward screen analyses.

While different approaches to tumor preparation and bioinformatic analyses can interfere with side-by-side comparisons of independent studies, so too does the combination of different SB components (e.g., the pairing of T2Onc2, T2Onc3, or GrOnc with *RosaSB* or *RosaSbas*e^*LSL*^) in the generation of a model (Figures 1A,B). Biologically this makes sense, as temporal onset, location, and kinetics of transposition are all influenced by transposon and transposase selection (2, 13, 18, 20). Indeed, transposon selection is the largest determinant of variability in candidate tumor driver identification among SB models of the same cancer type (20, 70). It is precisely this diversity and flexibility that make the SB system an indispensable tool in studying cancer genetics. The SB insertional mutagenesis system has been used to model numerous forms of cancer types and led to the identification of innumerable candidate drivers, many of which have been subsequently validated (reviewed elsewhere) (71, 72). Two publicly available databases, the Candidate Cancer Gene Database (CCGD) and the Sleeping Beauty Cancer Driver Database (SBCDDB), allow for streamlined searching of these candidate genes (73, 74). In the following paragraphs we explore variables that, when present, have the potential to influence tumorigenesis as reflected by alteration of the SB tumor driver landscape.

## Sensitizing Mutations in SB Mouse Models

Combining a predisposing (or sensitizing) mutation with the SB system can facilitate tumorigenesis. Sensitizing mutations are selected based on their known role as a tumor suppressor or oncogene in the cancer type being studied. In some cases, these mutations are required to induce cancer formation in an SB model (7, 22, 57, 75, 76). A comprehensive list of sensitizing mutations used in SB mouse models of cancer is presented in this review (Table 1). When tumorigenesis is observed in SB mice on both wildtype and sensitized backgrounds, investigators have the opportunity to compare differences in mutation profiles between the two cohorts. In this way, mutations driving tumor development on a wildtype background can be distinguished from genetic drivers co-occurring with a particular mutation. Such information allows for better tumor subtype stratification. Published comparative analyses conducted on screens of lymphomas, acute and chronic myeloid leukemias, osteosarcoma, and colorectal disease are discussed below.

**Table 1:** Sensitizing mutations used in SB cancer screens.

**Gene mutated**	**Tumor (or tissue) type and references**
*Apc*	Intestinal (77, 78)
*Blm*	Glioma (24)
*Braf*	Melanoma (7, 75, 76)
*Cadm1*	Leukemia/CD3-positive T-Cell lymphoma (predominant) (79)
*Ccne (cyclin E)*	Leukemia (erythroleukemia and T-ALL) (80)
*Cdh1*	Breast (invasive lobular phenotype predominant) (81)
*Csf*	Glioma (24)
*Ctnnb1*	Breast (82)
*Egfr*	Peripheral nerve sheath (4)
*Hras*	Skin (non-melanoma) (23)
	Thyroid (poorly differentiated predominant) (83)
*Jak2*	leukemia (erythroleukemia predominant) (84)
*Kras*	Intestinal (78)
	Pancreatic adenocarcinoma (22, 57)
*Myc*	Liver (85)
*Npm1c*	Leukemia (AML predominant) (21)
*p19arf*	Gliomas (24, 86)
	Lung (87)
	Multiple tumor types (13)
*Ptch*	Medulloblastoma (3, 88, 89)
*Pten*	Lung (predominant) (87)
	Prostate (90)
	Breast (91)
	Liver (92)
	Medulloblastoma (93)
*Rag2*	Multiple tumor types (94)
*Rassf1a*	Leukemia/poorly differentiated lymphoma (predominant) (95)
*Sav1*	Liver (6)
*Smad4*	Intestinal (78)
	Gastric adenomas (predominant) (96)
*Stat5b*	B-ALL (97)
*Tcl1*	CLL (98)
*Tgfbr2*	Intestinal (99)
*Trp53*	Liver (1)
	Leukemia/CD3-positive T-Cell lymphoma (predominant) (100)
	Lung (87)
	Medulloblastoma (3, 88, 101)
	Osteosarcoma (8)
	Breast (102)
	Lymphoma, B cell (103)
	CNS-PNET (93)
**Genes mutated** **(co-occurring)**	**Tumor (or tissue) type and references**
*BCR-ABL* (translocation)	Leukemia (CML) (104)
*Egfr* and *Trp53*	Peripheral nerve sheath (4)
*Etv6-RUNX1* (fusion)	Leukemia (BCP-ALL predominant) (105)
*Etv6-RUNX1* (fusion) and *Pax5*	Leukemia (BCP-ALL predominant) (106)

*BCP-ALL, B-cell precursor acute lymphoblastic leukemia; CNS-PNET, central nervous system primitive neuroectodermal tumor. AML, acute myeloid leukemia; B-ALL, B-cell acute lymphoblastic leukemia; CLL, chronic lymphocytic leukemia; CML, chronic myeloid leukemia*.

### Lymphomas

Lymphomas are cancers of the lymphatic tissues in the body. Three SB lymphoma publications studied SB-induced “lymphoma/leukemia” in the context of *Cadm1*-null, *Trp53* homozygous and heterozygous mutant, and *Rassf1*-null backgrounds (79, 95, 100). *TP53* and *RASSF1* are both tumor suppressors known to be inactivated in human lymphomas (107–110). *CADM1* belongs to the immunoglobulin superfamily of cell adhesion molecules. It was initially characterized as a tumor suppressor with loss of expression observed in various cancer types including hematologic malignancies (111, 112). More recently, cell surface expression of *CADM1* has been described as an indicator of disease status and progression in some lymphoma subtypes (113, 114).

In the first of their studies published in close succession, tumors from 117 *Cadm1*^−/−^; *SB* and 73 *Cadm1*^+/+^; *SB* littermates were found to be predominantly CD3-positive T-cell lymphoma (79). SB mice on the *Cadm1* null background had increased tumor multiplicity that developed with decreased latency (79). Ten genes including *Nr3c1*, the most frequently disrupted gene, were unique to the *Cadm1*-null background (79). Fifteen candidate genes were shared between the two cohorts while six genes were identified exclusively in *Cadm1*^+/+^; *SB* tumors. The SB insertion pattern observed in *Nr3c1*, the gene that encodes the glucocorticoid receptor, is suggestive of its role as a tumor suppressor (79). Authors noted that three other genes identified exclusively in the *Cadm1*-null SB cohort of tumors (*St13, Ets1*, and *Csf3r*) encode proteins that regulate or interact with the glucocorticoid receptor (79). Indeed, impaired glucocorticoid signaling, via various mechanisms including deletion of *NR3C1*, has been associated with relapse and poor prognosis in pediatric acute lymphoblastic leukemia and blastic plasmacytoid dendritic cell neoplasm (115–117). These data demonstrate a selective advantage for combining impaired cell adhesion and disrupted glucocorticoid signaling in lymphomagenesis. The mechanistic and clinical implications of this observation remain to be explored.

In the second forward screen performed in mice on a *Rassf1*-null background, 111 *Rassf1*^−/−^*; SB* and 25 *Rassf1*^+/+^; *SB* tumors were predominantly poorly differentiated lymphoma (95). Tumor latency was decreased in SB mice on the *Rassf1*-null background. Authors noted that their statistical analysis included data from an additional 101 SB mice on a wildtype background. These mice lacked the 129/Sv background introduced by breeding with *Rassf1*-null mice (95). Nevertheless, candidate drivers unique to the *Rassf1*-null background were identified including the transcription factor *Runx2* (95). When present, Rassf1 alters Hippo signaling by influencing the proteins with which Yap1 complexes while Runx2 complexes with Yap1 directly (118–120). Authors posited that loss of both *RASSF1* and *RUNX2* exacerbates YAP1-TEAD complex formation leading to increased cellular proliferation and supported this hypothesis with *in vitro* assays (95).

In the analysis of *Trp53*-related tumors, CD3-positive T-cell lymphoma was again identified as the predominant tumor type (100). Nine *Trp53*^−/−^, 116 *Trp53*^+/−^, and 36 *Trp53*^+/+^ SB-induced tumors were analyzed. Eight of the nine candidate genes identified in the *Trp53*^−/−^; *SB* group were unique to that group. Twelve genes were shared between *Trp53* heterozygous and *Trp53* wildtype SB cohorts. One gene, *Rapgef6*, was shared between both *Trp53*^−/−^; *SB* and *Trp53*^+/−^, *SB* cohorts but not detected in *Trp53*^+/+^; *SB* tumors (100). *Jdp2* disruption leading to overexpression was enriched in SB tumors maintaining *Trp53* heterozygosity (100). Jdp2 directly binds the *Trp53* promoter to represses its expression (100, 121). Thus, investigators concluded that *Jdp2* mutation facilitates tumorigenesis in *Trp53* heterozygotes by rendering a biallelic *Trp53* loss-of-function phenotype.

In another model, T2Onc and RosaSBase^LsL^ with or without conditional *Trp53*^*R*270*H*^ resulted in 65% of SB mice developing B cell lymphoid disease histologically compatible with follicular lymphoma or diffuse large B cell lymphoma (103). In an evaluation of 23 *SB*-only and 7 *SB*-*Trp53* tumors, investigators identified 48 and 12 candidate tumor drivers within each group, respectively. It is difficult to draw conclusions on influence of the predisposing *Trp53* mutation on mutational profile given the small sample size. Authors did, however, further evaluate the Ras-responsive element binding protein 1 (*Rreb1*) transcription factor identified in the *SB*-only tumors. *RREB1*'s influence on *KRAS* expression increased RAS/MAPK signaling *in vitro* and was found to be overexpressed in a subset of human diffuse large B-cell lymphomas (DLBCLs) (103).

### Acute Myeloid Leukemia (AML)

Mutations involving nucleophosmin (*NPM1*) are observed in ~60% of cytogenetically normal AML tumors (122). Vassiliou et al. studied the impact of heterozygous expression of a “humanized” version of the most common mutation, *Npm1*c^*A*^, on the transposon insertion profile of an SB model of AML (21). Tumors from 87 *Npm1*c^*A*/+^; *SB* mice and 34 *Npm1c*^+/+^; *SB* mice were assessed. Investigators found 75% (18/24) of candidate tumor drivers identified in the *Npm1*c^*A*/+^; *SB* tumors to be unique to that cohort (21). Cooperative mutations with *Npm1*c^*A*^ included SB insertion patterns consistent with activation of *Csf2* (observed in 48% of tumors) and *Flt3*. Both granulocyte-macrophage colony stimulating factor (*CSF2*) and the tyrosine kinase receptor *FLT3* are frequently mutated in human AML with *FLT3* mutations commonly co-occurring with *NPM1* mutations (123–125). The internal tandem duplication mutation of *FLT3* (*FLT3*-*ITD*) is known to cause constitutive activation of the JAK/STAT pathway (126). The synergism of co-mutated *Npm1* and *Flt3-ITD* in AML tumorigenesis was further demonstrated *in vivo* and corresponded with a pronounced overall change in lymphoid progenitor cell gene expression (127, 128).

### Chronic Myeloid Leukemia (CML)

The *BCR-ABL* fusion gene is generally considered a disease-defining mutation of CML. CML is a slow-growing tumor, with patients appropriately treated with a tyrosine kinase inhibitor targeting BCR-ABL remaining in the chronic phase for many years. Progression to the accelerated and blast crisis phases is characterized by acquisition of new chromosomal aberrations and increasing number of blasts. Once in blast crisis, CML behaves more like acute myeloid leukemia and median survival is ~12 months (129). Previously, mice engineered to express the disease-defining BCR-ABL translocation in hematopoietic stem cells were found to recapitulate chronic phase CML, with a small subset of tumors progressing to blast crisis (130). To further explore the genetics of CML progression, Giotopoulos et al. crossed the *BCR-ABL* mouse to mice harboring the GrOnc transposon and *Mx1*-*Cre* mediated activation of *RosaSbas*e^*LSL*^ SB system (*BCR-ABL; SB*) (104). The GrOnc construct contains the Graffi1.4 murine leukemia virus LTR, which preferentially promotes myeloid lineage cells (131). Authors also bred *SB-*only mice lacking the *BCR*-*ABL* translocation. Phenotypic and microarray gene expression comparisons between the *BCR-ABL* and *BCR-ABL; SB* cohorts were conducted. *BCR-ABL; SB* mice were found to have decreased survival, indicators of disease progression (increased terminal WBCs, decreased hemoglobin, increased spleen and liver weights), and changes in expression of genes previously implicated in CML progression when compared to *BCR-ABL*-only mice (104). Microscopic and immunophenotypic evaluation of tumors revealed all *BCR-ABL*-only mice to have disease that remained in the chronic phase. *SB*-only mice developed both lymphoid (26%) and myeloid (70%) acute leukemias. *BCR-ABL; SB* mice also developed primarily myeloid acute leukemia (85%). Interestingly, the *BCR-ABL; SB* cohort was the only group to manifest a continuum of CML progression, with tumors in the intermediate accelerated phase (10%) and blast crisis phase (5%) (104).

Candidate tumor driver analysis conducted on tumor DNA from 52 *BCR*-*ABL*; *SB* mice and 20 *SB*-only mice revealed 78/91 (86%) of candidate drivers identified in the *BCR-ABL; SB* tumors to be unique to that cohort (104). Authors noted that, among genes identified, several had already been implicated as potential drivers of CML and CML progression including: *Asxl1, Myb, Stat5b*, and *Pten* (104). Genes not previously associated with CML progression included: *Jak1, Flt3, Nf1, Erg*, and *Mll3* (104). Authors found the ETS-related gene (*Erg*) transcription factor to be most frequently disrupted, with an overall transposon insertion profile suggestive of oncogenic activation (104). To further assess its role *in vivo, ERG* was overexpressed using a retroviral vector in hematopoietic stem cells derived from *BCR*-*ABL*-expressing and wildtype mice. Cells were then transplanted into congenic recipient mice (104). The *ERG*; *BCR*-*ABL* mice developed acute leukemias phenotypically resembling the *BCR*-*ABL*; *SB* mice. Overexpression of *ERG* on a *BCR*-*ABL* background significantly decreased survival relative to *ERG* on a wildtype background and *BCR*-*ABL*-only mice. Authors noted that ERG has been implicated in poor prognosis and blast-phase transformation of other hematological diseases (104). As more data regarding chromosomal and gene expression changes in CML progression are obtained, clarity as to the clinical relevance of *ERG* in CML may begin to emerge.

### Osteosarcoma

The third most common cancer among children and young adults, osteosarcomas harbor pronounced chromosomal aberrations (132). A limited number of genes are implicated in tumorigenesis, one of which is *TP53*. In an SB model of osteosarcoma, introduction of the *Trp53*^R290H^ dominant negative allele accelerated tumorigenesis and increased tumor burden and penetrance relative to *SB*-only mice and *Trp53*-only mutant mice (8). Mice from both *SB* cohorts (with and without the *Trp53* mutant) developed liver and/or lung metastases (8). Comparative genomic hybridization and karyotyping revealed *SB*-only tumors had fewer genomic aberrations relative to *Trp53*; *SB* tumors. In a comparison of transposon insertions from 96 *Trp53*; *SB* and 23 *SB*-only osteosarcomas, Moriarity et al. observed overlap of the top hits in both cohorts including: *Pten, Eras*, and *Nf1* (8). Investigators validated the cooperative nature of the combined *Pten* and *Trp53*^*R*270*H*^ mutations *in vivo* (8). Mutation of *TP53* and loss of *PTEN* are frequent derangements in human osteosarcoma, with loss of *PTEN* associated with a poorer clinical prognosis (133, 134). Indeed, in a recent whole exome phylogenetic analysis of osteosarcomas, mutation of *TP53* was determined to be an early event while loss of *PTEN* was associated with lung metastases (135).

### Colorectal Disease

Starr et al. compared candidate tumor drivers of colorectal disease (predominantly adenomas) identified in heterozygous *Apc* (*Apc*; *SB*) model with those from *SB*-only tumors (77, 136). Investigators found three genes (fewer than 4%) identified in the *Apc*; *SB* screen to also be affected in the *SB*-only screen (77). The shared genes were *Apc* (resulting in biallelic inactivation in *Apc*; *SB* mice), *Nsd1*, and *Wac*. Authors noted 70% of candidate drivers from the *Apc*; *SB* tumors had one or more transposon insertions within the same locus in the *SB*-only tumors although these insertions did not occur at a rate high enough to be considered candidate drivers in *SB*-only tumors using their conservative statistical approach (77).

In another SB model of intestinal disease, Takeda et al. compared the candidate tumor drivers identified in tumors from mice with one of three predisposing mutations: *Kra*s^*G*12*D*/+^, *Smad4*^*KO*/+^, or *Trp53*^*R*172*H*/+^ with a pooled list of candidate drivers identified in SB tumors from mice with somatic or germline *Apc* mutations (collectively referred to as *Apc*) (68, 78). Investigators found ~50% overlap in candidate genes identified in the three cohorts of tumors with the previously published *Apc* list (78). One hundred and eleven genes (~8% of all identified candidate tumor drivers) were disrupted in all four groups (78). While *Apc* disruption was the most prevalent event in *Apc-, Kra*s^*G*12*D*^-, and *Trp53*^*R*172*H*^*; SB* tumors, only 32% of *Smad4*^*KO*/+^*; SB* tumors demonstrated insertional disruption of *Apc* with the majority of tumors (79%) displaying biallelic inactivation of *Smad4* (78). Upregulation of Wnt/B-catenin signal activating R-spondin genes (*Rspo1* or *Rspo2*) via transposon insertion was also significantly enriched in *Smad4*^*KO*/+^; *SB* tumors. While these comparisons suggest sensitizing mutations may uniquely influence tumor driver profiles, conclusions should be tempered by the fact that strain backgrounds varied among the mice, introducing additional genetic variability (78).

Most recently, SB mutagenesis was used to identify genes cooperating with loss of TGF-B in intestinal neoplasms (99). Tumors from SB mice with homozygous conditional inactivation of TGF-B receptor, type II (*Tgfbr2*) were compared with tumors from SB mice on a wildtype background. Authors found 34% (232/673) and 50% (187/372) of candidate genes identified in *Tgfbr2; SB* tumors to be unique to the presence of *Tgfbr2* inactivation, depending on statistical method used (99). Comparison of the two statistical approaches revealed overlap of 17 genes, with an enrichment in genes responsible for either Wnt/B-catenin or Hippo pathway signaling including *Lrp6, Ppp2r1a, Tcf7l2*, and *Yap1* (99). Given the role of SMAD4 in TGF-B signaling, candidate genes identified in *Smad4*^*KO*/+^*; SB* tumors were compared to candidate genes in *Tgfbr2*; *SB* tumors with the expectation that significant overlap would be observed (78, 99). Indeed, authors found a 54% (243/449) overlap in the genes identified in the *Smad4*^*KO*/+^; *SB* screen and the *Tgfbr2*; *SB* screen with a decrease in the number of independent tumors harboring *Apc* transposon insertions relative to wildtype (27% (35/130) vs. 45% (58/130), respectively) (99).

Collectively, these studies reveal the dramatic influence single gene modification can have on tumor gene recruitment and overall tumorigenesis. While some candidate genes appear in more than one context, the identification of candidate tumor drivers unique to particular mutant backgrounds suggests selective gene cooperation in the formation of these tumor subtypes.

## Other Factors Influencing Tumor Development in SB Models

Manipulation of the tumor microenvironment can extend beyond the introduction of a sensitizing mutation. Known risk factors can be incorporated into study designs to interrogate their influences on tumor genetics. To date, investigators have used SB mouse models to explore genetic mediators of drug resistance, virus-associated disease, sex-biases, and immune function in cancer.

### Therapeutic Interventions

Patients with melanoma positive for the *BRA*F^*V*600*E*^ point mutation have overactivation of the MAPK pathway and, consequently, inhibition of apoptosis and uncontrolled cellular proliferation. In advanced disease, patients are candidates for the targeted protein kinase inhibitor vemurafenib (PLX4720). The initial clinical response is typically quite strong, but patients rapidly relapse and experience aggressive disease progression (137). SB technology has been utilized to identify potential mediators of vemurafenib monotherapy resistance in melanoma (75). In the study, mice harboring a melanocyte-specific 4-hydroxytamoxifen (4-OHT)-inducible *Tyrosinase*-*CreE*R^*T*2^ allele and inducible mutant *Braf* allele in the endogenous *Braf* locus (*Tyr*-*Cr*e^*ERT*2^; *Bra*f^*lsl*−*V*618*E*/+^) were mated with *SB* mice (offspring hereafter referred to as *Braf*-*SB* mice). After application of 4-OH tamoxifen and expression of oncogenic *Braf* and the *SB* system, *Braf*-*SB* mice were aged and monitored for tumor development. Upon melanoma formation, a subset of the mice were administered a diet containing vemurafenib. Tumors in these mice were observed to regress over a 1–4 week period followed by a period of stable disease and subsequent relapse marked by tumor regrowth/progression (75). Genomic DNA from treatment-naïve and vemurafenib-resistant melanomas was harvested and candidate tumor drivers identified. Statistical comparison of genes identified in treatment-naïve and vemurafenib-resistant melanomas revealed eight candidate tumor drivers enriched in treatment-resistant tumors: three known mediators of vemurafenib resistance (*Braf* , *Mitf* , and *Cdkn2a*) and five novel candidate genes (75). Authors validated one of these novel candidate genes, *ERas*, in a human cell line assay (75).

*ERAS* (embryonic stem cell-expressed Ras) encodes a constitutively active RAS-like protein that potentiates PI3K/AKT signaling. Crosstalk between MAPK and PI3K/AKT signaling is well-established, with PI3K/AKT upregulation a recognized mechanism by which vemurafenib resistance can occur [recently reviewed by (138)]. In their experiments, Perna et al. demonstrated the ability of ERAS to evade vemurafenib-mediated inhibition of the MAPK pathway and consequent dephosphorylation/activation of BAD, a proapoptotic protein, by promoting inactivation of BAD via the AKT pathway (75). Indeed, a phase I/II clinical trial of for the dual treatment of patients with *BRAF*-positive advanced melanoma with BKM120, a PI3K inhibitor, and vemurafenib had been initiated, although terminated early due to therapy-related toxicity (NCT01512251). Nevertheless, these data demonstrate the clinical relevance of SB screens in identifying possible genetic mediators of drug resistance and underscore their potential to inform future clinical research and treatment.

### Environmental Exposures

Chronic HBV infection is known to increase the risk of hepatocellular carcinoma (HCC) and is attributable to half of all HCC cases (139, 140). Mice engineered to express the HBV surface antigen (HBsAg) develop hepatocellular inflammation, hepatic necrosis, regeneration, benign adenomas, and subsequent HCC (141). Two different SB models with liver-specific activation of the SB system (one utilizing the T2Onc2 transposon, the other using T2Onc3) have been deployed in the presence of HBsAg activation to identify candidate drivers of tumor formation in the context of HBV-induced inflammation (6, 142). Although a detailed comparison was not made, Kodama et al. observed a high degree of concordance between candidate tumor drivers identified in SB tumors with HBsAg-induced inflammation and SB alone after pooling data from both T2Onc2 and T2Onc3 screens (142). Hepatic fibrosis and cirrhosis are common features of chronic HBV infection in humans. Indeed, 90% of all HCCs develop in the context of cirrhosis (143). It is important to note, however, that no overt fibrosis is observed in the HBsAg mouse model (6, 141).

To directly assess the influence of a fibrotic microenvironment on HCC formation, Riordan et al. evaluated tumor formation in the context of chemical-induced fibrosis in a liver-specific SB mutagenesis model (*Al*b^*Cre*/+^*; SB*) (70). Two lines of T2/Onc3 transposon mice (TG12740 and TG12775) were used to permit full genome coverage. Chronic fibrosis was induced in 77 animals via intraperitoneal injection of carbon tetrachloride (CCl4). Tumors developed in both treated and untreated mice, permitting evaluation of the impact of fibrosis on tumor driver landscape. Twenty-one genes were identified as candidate tumor drivers in both cohorts including the most frequently mutated gene in both cohorts, *Rtl1* (70). Certain drivers were more common in the CCl4-treated cohort of tumors including: *Gli2, Fign*, and *Met*, suggesting a propensity for their disruption in the context of fibrosis (70). Authors performed an *in vivo* validation of *Gli2*, a transcription factor activated by Hedgehog pathway signaling, in the context of fibrosis (70). Authors also validated *Fign*, which was mutated in 8% (27/343) of CCl4 tumors and none of the SB-only tumors, in an *in vitro* invasion assay (70). Interestingly, in a comparison of genes mutated in ≥2% of CCl4 tumors with those mutated in ≥2% of tumors from previously published HBsAg models, authors found 88% (50/57) overlap, suggesting an enrichment of these tumor drivers in the context of chronic hepatocellular injury.

Other investigators have assessed the impact of steatosis on hepatocellular disease (92, 144). Tschida et al. described an SB model of hepatic tumor formation in the context of alcohol-induced steatosis (144). SB mice harboring the T2Onc transposon and *Al*b^*Cre*/+^-mediated induction of *RosaSbas*e^*LSL*^ were administration ethanol and a choline-deficient diet (144). In their model, 37/49 (76%) of SB mice receiving the treatment diet developed well-differentiated adenomas (81%) or HCC (19%) (144). Authors compared the transposon insertion profiles of tumors collected from 36 steatosis-induced SB mice (15 males and 21 females) to the insertion profile previously reported in tumors from SB mice harboring the same SB components on a *Trp53*-deficient background administered a normal diet (1). While this comparison is not ideal given the predisposing *Trp53* mutation in one group and not the other, investigators chose to pursue *in vivo* analyses of two candidate genes enriched in tumors from the steatosis cohort, *Prkaca* and *Nat10*. Vectors expressing either constitutively active *Prkaca* (*Prkac*a^*L*206*R*^), fumarylacetoacetate hydrolase (*Fah*), and short hairpin RNA targeting *Trp53* (sh*Trp53*) or *Fah* and sh*Trp53* alone were introduced into *Fah*-deficient male and female mice on either the steatosis-inducing treatment diet or normal diet. Mice in both the treatment and normal diet groups injected with dual *Prkac*a^*L*206*R*^ and sh*Trp53* demonstrated higher tumor burden and penetrance relative to *Trp53* alone (144). Moreover, a higher percentage of dual *Prkac*a^*L*206*R*^/sh*Trp53* tumors were histologically classified as HCC relative to sh*Trp53* alone (14 and 0%, respectively). Among mice on the ethanol and choline-deficient steatosis-inducing diet, those receiving dual *Prkac*a^*L*206*R*^/sh*Trp53* demonstrated higher tumor penetrance and burden relative to *Prkac*a^*L*206*R*^/sh*Trp53* mice on normal diet, suggesting a tumorigenic role for *Prkac*a^*L*206*R*^ in the context of hepatic steatosis (144). The same study design was executed using a *Nat10* vector. Authors found no difference in tumor penetrance in *Nat10*/sh*Trp* mice on treatment diet vs. normal diet.

Utilizing a similar study approach, Kodama et al. compared the genetic profiles of hepatocellular tumors developing in the context of steatosis induced by one of two mechanisms: high fat diet or homozygous *Pten* loss (92). For their study, investigators utilized T2Onc2 mice with *Al*b^*Cre*/+^-mediated induction of *RosaSbas*e^*LSL*^. Again, while utilization of the *Pten* mouse strain introduced genetic variability between the two groups that is difficult to account for (the SB mice were crossed with the C;129S4-*Pte*n^*tm*1*Hwu*^/J strain), investigators found 10/30 (33%) of candidate tumor drivers making up ≥2% of total reads in tumors from the high fat diet group to overlap with those identified in the *Pten* group. One of these genes, *Sav1*, had been previously used to predispose for cancer in an SB model but had not been identified as a candidate driver in an SB hepatocellular screen (6). Authors found *Sav1* loss to accelerate hepatocellular carcinoma in the context of *Pten*-deficient steatotic liver *in vivo* (92). These results were replicated by another group (145).

Indeed, biologically relevant connections between novel SB candidate hepatocellular tumor drivers and cellular responses to damage exist, strengthening the hypothesis that these genes mediate tumorigenesis in the context of hepatocellular injury. Candidate genes *Gli2, Fign*, and *Sav1* are further discussed here.

Gli2 is a transcription factor activated by Hedgehog (Hh) signaling. While the Hh pathway is not active in normal hepatocytes, it can be induced in response to certain cellular stressors (146). In a mouse model of renal fibrosis, mice with myofibroblast-specific *Gli2* deletion demonstrated cell cycle arrest and reduced fibrosis after ureteral obstruction (147). The same authors showed the ability of darinaparsin, a *GLI2* inhibitor, to both prevent and ameliorate fibrosis in wildtype mice exposed to ureteral injury (147). The mechanism by which *Gli2* may mediate hepatocellular cancer is less clear, although it is possible that overexpression is oncogenic by virtue of its ability to induce fibrosis and perpetuate cellular proliferation.

*Fign* encodes an ATP-dependent microtubule severing protein. Hepatocytes rely heavily on the microtubule cytoskeleton to maintain polarity and traffic proteins to appropriate regions of the cell. Indeed, hepatocytes are uniquely multipolar, with each cell interfacing with multiple bile canaliculi and endothelial surfaces carrying sinusoidal or portal venous blood (148). Alpha-tubulin, one of the primary proteins making up the microtubule structure, is known to undergo post-translational modifications upon exposure to ethanol metabolites (149). These modifications have been shown to decrease intracellular trafficking (149). In their review, Groebner and Tuma hypothesize that ethanol-induced microtubule modifications lead to altered lipid droplet transport, thereby contributing to the steatotic liver disease phenotype associated with ethanol consumption. *Fign* may function in a similar way, sufficiently disrupting microtubule stability to facilitate hepatic fibrosis and oncogenesis.

Sav1 is a scaffolding protein involved in the Hippo signaling pathway. Expression of *Sav1* leads to the phosphorylation and degradation of the transcription regulators Yap and Taz (150). Deletion of *Sav1* in the murine liver causes hepatomegaly and cancer after a latency of 1 year (145, 151). Mice with *Pten* deletion manifest a fatty liver phenotype prior to the development of cancer that also occurs after a prolonged latent period (92, 145, 152). Combined *Sav1* and *Pten* deletion not only accelerates HCC formation, but increases hepatic steatosis via synergistic activation of molecules downstream of Hippo and Pi3k signaling (145).

Divergences in findings among SB models of HCC underscore the significance of genetic and/or experimental variability in influencing tumorigenesis. Nevertheless, in assessing candidate tumor genes identified across SB models of hepatocellular disease, investigators have observed substantial overlap, suggesting an enrichment for particular tumor drivers in the context of chronic hepatocellular injury (70, 92). Further use of these models in the presence of specific types of hepatic injury (viral, alcoholic, fibrotic, steatotic) may increase the precision with which we are able to tease out differences and inform how we manage discrete patient populations.

### Sex Bias

Male sex is a known risk factor for HCC across populations, with male to female incidence ratios averaging between 2:1 and 4:1 (153). Mouse models of HCC, including those utilizing the SB system, also demonstrate sexual dimorphism (1, 6, 20, 141). Keng et al. sought to identify sex-specific genetic drivers of HCC by comparing transposon insertion profiles of hepatic lesions occurring in a small cohort of male and female mice (154). Data from both sexes were pooled from mice harboring liver-specific activation of the SB system alone or in conjunction with *Trp53* inactivation for a total of 10 male and 9 female mice (154). Authors noted that while the majority of lesions harvested from males were classified as HCC, female nodules were histologically characterized as premalignant dysplastic lesions, adenomas, or HCC. *Egfr* was identified in nodules from all male mice evaluated (10/10), while only 22% of female mice (2/9) had tumors with *Egfr* identified as a candidate tumor driver (154). Genetic data from human HCC samples corroborated gender-specific findings, as tumors with polysomy of chromosome 7 (the location of human *EGFR*) displayed elevated *EGFR* mRNA expression and were disproportionately male (33:1 male to female ratio in the polysomy 7 subclass vs. 2:1 ratio in all other subclasses of human HCC evaluated) (154). Furthermore, authors performed an *in vivo* validation of EGFR by introducing expression vectors of truncated *EGFR* and short hairpin RNA targeting *Trp53* into male and female *Fah*-deficient mice (154). Male mice were found to have a significantly increased number of hepatic lesions per mouse relative to female mice (154). Lesions in both sexes were categorized as HCCs by histological evaluation (154). Intriguingly, Tschida et al. reported a reduction in sexual dimorphism in their SB model of hepatic tumor formation in the context of steatosis, a trend also observed among human HCC cases when stratified by steatosis status (144).

Pronounced sex differences are known to exist in terms of hepatic response to toxins, including alcohol and viruses. These differences are, at least in part, attributable to differential expression of sex steroids and their influences on pathways involved in inflammation, lipid metabolism, and insulin response (155), (156–158). Indeed, crosstalk between steroids and EGFR is known to occur and baseline *Egfr* expression in normal mice differs between males and females (159). In a recent analysis of genetic data from The Cancer Genome Atlas (TCGA), male liver tumors were found to exhibit higher densities of single nucleotide variants (SNVs) and lower expression of mismatch repair (MMR) genes compared to female liver tumors (160). This trend was observed in several tumor types. Indeed, increased expression of *EGFR* mediates inhibition of MMR and facilitates error-prone DNA replication (161). As such, SB mouse models of cancer are able to recapitulate trends observed in human populations, reaffirming their potential utility in understanding tumorigenesis in humans.

### Immune Function

In recent years, the concept of immunosurveillance as a force shaping tumor biology has emerged (162, 163). Therapies including immune checkpoint modulators, immune cell therapy, and immune-modifying agents are proving highly effective in inducing tumor regression in patients (164). Despite advances, genetic drivers of tumor immunogenicity and immune system evasion are poorly understood. Rogers et al. sought to characterize the impact of the adaptive immune system on tumorigenesis in an SB model of cancer (94). The *Rag2* deficient (*Rag2*^−/−^) mouse is unable to produce mature B and T cells and has an increased incidence of spontaneous and carcinogen-induced cancers (165). Moreover, immunological control of tumor development has been demonstrated in *Rag2*^−/−^ mice exposed to a chemical carcinogen (166). In their experiment, Rogers et al. bred immunocompromised (*Rag2*^−/−^) and immunocompetent mice (*Rag2*^+/−^) to mice with ubiquitously activated SB. Tumor latencies and multiplicities were similar in *Rag2*^−/−^*; SB* and *Rag2*^+/−^*; SB* mice (94). Authors offered various explanations for this including the possibility that SB-induced mutations, which cause altered gene expression rather than the creation of strong, highly mutated antigenic targets, result in less immunogenic tumors (94). Nevertheless, the genetic profiles of two out of three cancer types evaluated revealed stark differences. The E1 ubiquitin-conjugating enzyme *Uba1* was mutated exclusively in leukemias from immunocompromised mice. Clonal insertions were also observed in skin tumors and HCC from *Rag2*^−/−^; *SB* mice (94). HCC tumors from mice with intact adaptive immune systems (*Rag2*^+/−^*; SB*) had insertions in *Rtl1* at a higher frequency than HCCs harvested from immunocompromised mice (94).

That *Uba1* was exclusively mutated in tumors that developed in the absence of an adaptive immune response suggests it is only an effective tumor driver in an immunocompromised microenvironment. A possible corollary to this is that *Uba1* offers no tumorigenic advantage in an immunocompetent environment. It is feasible that mutation of *Uba1*, a ubiquitinase also known to be involved in protein folding and degradation, leads to the generation of innumerable antigenic peptides that facilitate clearance of the tumor cells in the presence of functional lymphocytes. Conversely, the predisposition for clonal selection of *Rtl1* in an immunocompetent environment suggests it assists in immune evasion. *Rtl1* is a retrotransposon-derived imprinted gene. It is expressed during development and is integral to maintaining the maternal-fetal interface of the placenta (167). Placental development involves marked cellular proliferation, invasion of the uterine wall, and successful evasion of the maternal immune system. Thus, clear biological and functional parallels between the placenta and cancers exist (168). Conceptually, it is within reason that *Rtl1*-mediated tumorigenesis may, at least in part, be due to a return to a fetal cell phenotype lacking immunogenic antigen expression.

These studies demonstrate that tumor microenvironments not only impact tumor incidence and kinetics, but also selection of driver mutations, supporting the hypothesis that complex genetic heterogeneity observed in human tumors could be explained by microenvironmental factors. Knowledge of context-specific genes driving tumorigenesis could guide treatment decisions in cancer care.

## The Future of SB Mutagenesis Screens

The utility of the SB system is manifest in its capacity to induce and tag mutations driving tumorigenesis in a targeted, streamlined, and unbiased manner. The simplicity of the SB system offers an ideal platform from which to make complex inquiries into cancer biology. Future modeling of SB cancers in unique genetic and microenvironmental milieus will provide a more sophisticated understanding of cancer genetics. Future directions in SB study design are explored below.

### Therapeutic Interventions and Environmental Exposures

**Figure 3:**
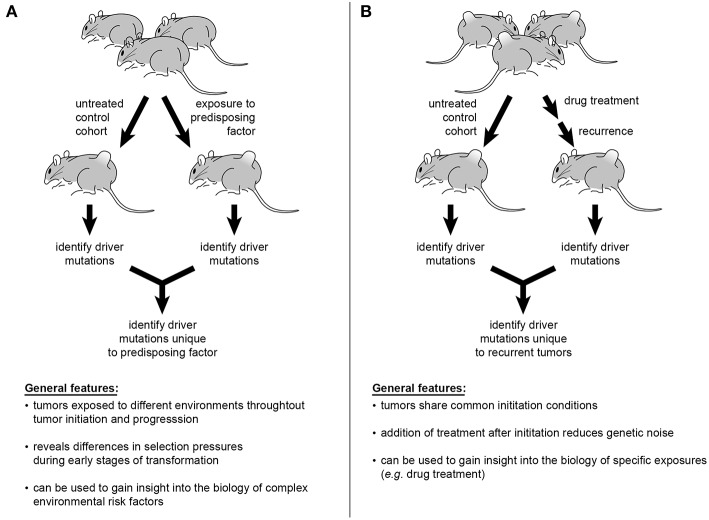
Sleeping Beauty mouse models can be used to interrogate a variety of influences on cancer genetics. Incorporation of epidemiologically described risk factors into study design offers the ability to identify the genetic alterations mediating tumorigenesis in at-risk populations **(A)**. The multiplexing of various pharmacological therapies used in the treatment of cancer patients would permit tracking of genetic changes leading to disease recurrence and progression **(B)**.

Despite epidemiological data establishing correlations between certain exposures, or “risk factors,” and cancer, the mechanistic complexities mediating such risks render the biology of these relationships difficult to understand. As an example, GWAS studies suggest numerous alleles influence the link between obesity and increased risk of endometrial cancer (169, 170). Incorporation of obesity into an SB model of endometrial cancer by providing a high-fat diet during or prior to SB activation would permit comparison of insertional profiles between obese and normal weight mice. Such interrogations could be conducted with a plethora of environmental factors including, but not limited to, UV exposure and skin cancers, pesticides and leukemias, or androgens and prostate cancer (Figure 3A). Multi-drug treatments and resulting relapses could also be systematically explored. To interrogate modulations in immune function, factors such as illness or pharmaceutical intervention (e.g., transplant anti-rejection drugs or HAART therapy) could be directly incorporated into experimental models to interrogate effects on tumor genetics (Figure 3B) (171, 172). Recently, the ability of experimental mice, housed in clean, low-pathogen facilities, to accurately recapitulate human immune responses has been brought into question (173). This is an intriguing idea in light of the enormous amount of scientific data emerging on the interplay among pathogens, the microbiome, and human physiology. Furthermore, preliminary studies suggest that pathogen exposure leads to maturation of the mouse immune response, with the genetic expression profile of peripheral mononuclear cells more closely resembling that of adult humans (174, 175). In the presence of such biologically relevant exposures, investigators may be able to better understand the biology of current treatment challenges and therapeutic roadblocks.

### Inherent Traits

Inbred mouse strains allow scientists to control for confounding genetic variability in experiments. While essential to establishing causality of a specific mutation or exposure, it removes population diversity. The incidence of spontaneous and carcinogen-induced cancers is known to vary significantly among inbred strains, underscoring the importance of genetic background and polymorphisms on tumorigenesis (176–180). Exploiting polymorphisms in genetically engineered mouse models of cancer may facilitate characterization of cancer phenotypes observed in human populations (181). Twin studies have shown that, for some spontaneous cancers, heritability contributes to causation by 27–42%-higher than that accounted for by known single-gene familial cancer syndromes (182). Indeed, while influence of germline polymorphisms on cancer risk is recognized, the impact of germline alleles on driver mutation selection is far less clear. Since introduction of a single sensitizing mutation can influence candidate tumor driver selection (as discussed in the “Sensitizing mutations in SB mouse models” section above), we speculate that altering the entire genetic background could have profound effects on SB candidate tumor driver profiles. Experimental designs could be conducted using mice from different strains engineered to harbor the SB system and mutational landscape assessed. Differential findings in candidate tumor drivers could then be compared to known allelic variants between strains.

### Genetics of Metastasis

Despite metastasis being the primary source of morbidity and mortality for many cancer types, the genetic drivers of metastasis are poorly understood. Moreover, the inherent complexity of tumors limits our ability to detect novel drivers of metastasis even when patient primaries and matched metastases are available for deep sequencing (183–185). In SB models, few shared transposon insertions is indicative of an early seeding event and independent tumor development while many shared insertions suggests clonal evolution of the primary tumor with subsequent dissemination. To date, comparative analyses have been conducted on metastatic lesions from SB models of medulloblastoma and osteosarcoma (3, 8). Wu et al. found little overlap between medulloblastoma primaries and metastases. The presence of known oncogenes such as *Notch2* and *Tert* in primary tumors and not metastatic lesions led authors to conclude that dissemination was an early event, occurring prior to the development of mutations in these genes (3). Retroviral vector assays have been used to validate *Eras, Lhx1, Ccrk*, and *Akt* as mediators of medulloblastoma dissemination/metastasis *in vivo* (3, 186). Moriarity et al. observed transposon insertional profiles in osteosarcoma that suggested a heterogeneous nature of metastasis (8). While 65% of genes (43/66) were unique to metastatic lesions, analysis of mice with three or more lesions suggested dissemination occurred at different points of tumorigenesis in different mice. Indeed, two mice appeared to have more than one metastatic seeding event (8). In future studies, the ability to turn off SB transposition, such as through introduction of another recombinase within the SBase, could provide information on the temporality of metastasis and the genes driving it. Techniques such as laser microdissection or single cell sorting could also refine our understanding of tumor heterogeneity and metastasis (72, 187).

Ultimately, SB models of cancer are capable of interrogating microenvironmental factors in a multiplexed fashion that more closely approximates conditions under which human cancers develop and spread. SB experiments conducted to date reveal the tumor microenvironment to have pronounced ramifications on the genetic evolution of various tumor types. Continued application of the SB model to explore genetic heterogeneity of tumorigenesis promises to influence future therapeutic advancements in the treatment of human cancers.

## Author Contributions

AG-Y and CF performed the literature review. AD and AG-Y designed the figures. AG-Y wrote and all co-authors edited the manuscript.

### Conflict of Interest Statement

The authors declare that the research was conducted in the absence of any commercial or financial relationships that could be construed as a potential conflict of interest.
